# Mass spectrometry analysis of human P2X1 receptors; insight into phosphorylation, modelling and conformational changes

**DOI:** 10.1111/jnc.12012

**Published:** 2012-10-11

**Authors:** Jonathan A Roberts, Andrew R Bottrill, Sharad Mistry, Richard J Evans

**Affiliations:** * Department of Cell Physiology & Pharmacology, University of LeicesterLeicester, UK; † Protein and Nucleic Acid Chemistry Laboratory, University of LeicesterLeicester, UK

**Keywords:** ATP, binding, electrophysiology, mutagenesis, oocytes, purification

## Abstract

Recombinant FlagHis_6_ tagged Human P2X1 receptors expressed in HEK293 cells were purified, digested with trypsin and analysed by mass spectroscopy (96% coverage following de-glycosylation and reduction). The receptor was basally phosphorylated at residues S387, S388 and T389 in the carboxyl terminus, a triple alanine mutant of these residues had a modest ∼ 25% increase in current amplitude and recovery from desensitization. Chemical modification showed that intracellular lysine residues close to the transmembrane domains and the membrane stabilization motif are accessible to the aqueous environment. The membrane-impermeant cross-linking reagent 3,3′-Dithio*bis* (sulfosuccinimidylpropionate) (DTSSP) reduced agonist binding and P2X1 receptor currents by > 90%, and modified lysine residues were identified by mass spectroscopy. Mutation to remove reactive lysine residues around the ATP-binding pocket had no effect on inhibtion of agonist evoked currents following DTSSP. However, agonist evoked currents were ∼ 10-fold higher than for wild type following DTSSP treatment for mutants K199R, K221R and K199R-K221R. These mutations remove reactive residues distant from the agonist binding pocket that are close enough to cross-link adjacent subunits. These results suggest that conformational change in the P2X1 receptor is required for co-ordination of ATP action.

Extracellular ATP plays a signalling role through the activation of cell surface ionotropic P2X and metabotropic P2Y receptors (Burnstock 2006a). Seven mammalian P2X receptor subunits (P2X1-7) have been identified with intracellular amino and carboxyl termini, two transmembrane segments and a large extracellular ligand binding loop. The subunits come together to form homo- and hetero-trimeric cation channels with a range of properties (Surprenant and North 2009). At least one form of P2X receptor is expressed in almost all cell types at least at some stage during development. For example, P2X1 receptors are expressed on smooth muscle, platelets, immune and glial cells (Mulryan *et al*. 2000; Hechler *et al*. 2003; Lalo *et al*. 2008; Lecut *et al*. 2009). It is now clear that P2X receptors make a significant contribution to many physiological processes ranging from inflammation to muscle contraction and are targets for analgesic and anti-thrombotic drugs (Burnstock 2006b; Surprenant and North 2009).

The recent apo and ATP bound zebrafish P2X4 receptor crystal structures have provided a quantum leap in understanding of the receptor (Kawate *et al*. 2009; Hattori and Gouaux 2012) and substantiated previous work regarding stoichiometry, ionic permeation and residues involved in mediating ATP action (Young 2009; Browne *et al*. 2010; Evans 2010). However, although mutagenesis studies identified the conserved residues in the binding pocket important for co-ordination of the agonist the novel folded ‘U’ conformation of ATP in the crystal structure was not predicted from modelling as molecular docking algorithms are weighted towards established binding poses and interactions. The ATP bound zebrafish P2X4 receptor structures show significant conformational change in the extracellular loop on agonist binding (Hattori and Gouaux 2012) consistent with recent studies on P2X1 and P2X2 receptors (Kawate *et al*. 2011; Jiang *et al*. 2012; Lorinczi *et al*. 2012; Roberts *et al*. 2012). Cross-linking of P2X1 receptor subunits with 3,3′-Dithio*bis*(sulfosuccinimidylpropionate) (DTSSP) was used to show the trimeric nature of the receptor (Nicke *et al*. 1998) and would be expected to constrain channel movement; however, whether this had an effect on channel function was not tested. Determining the extent of DTSSP modification would give insight into the native P2X1 receptor structure, and potentially conformational movement.

The intracellular regions of the P2X receptor make a significant contribution to determining channel properties. Characterization of splice variants and a range of mutagenesis studies have shown channel gating is regulated by both the amino and carboxyl termini (e.g. Brandle *et al*. 1997; Smith *et al*. 1999; Boue-Grabot *et al*. 2000). There are several threonine, serine and tyrosine residues in the intracellular regions that are potential sites for phosphorylation. The receptors are sensitive to regulation by G-protein coupled receptors that stimulate protein kinase C (an amino terminal consensus site for this kinase is present in all the currently cloned P2X receptors) (Surprenant and North 2009), and tyrosine phosphorylation of the carboxy terminus has been shown to regulate P2X7 receptors (Adinolfi *et al*. 2003). The P2X receptors have a conserved sequence (YXXXK) in the carboxy terminus that constitutes a trafficking motif involved in stabilization of the receptor at the cell surface (Chaumont *et al*. 2004). Studies on P2X1 and P2X2 receptors have shown that lysine residues within the trafficking motif were involved in phospholipid binding to GST-carboxyl termini fusion proteins and phospholipid depletion regulated P2X receptor gating (Fujiwara and Kubo 2006; Bernier *et al*. 2008b). Taken together this suggests that the carboxy terminus trafficking region may be anchored to phospholipids in the leaflet of the membrane. The structures of the zfP2X4 receptor were solved in the absence of the intracellular regions that were removed to aid in crystallization (Kawate *et al*. 2009; Hattori and Gouaux 2012). Thus, it remains unknown the extent of folding/accessibility or phosphorylation of the intracellular domains.

We have recently used purified full length C-terminally tagged human P2X1 receptors to show agonist induced conformational change in single particle electron microscopy studies and identified a range of P2X1 receptor-interacting proteins (Lalo *et al*. 2011; Roberts *et al*. 2012). In this study, we have used mass spectrometry of purified P2X1 receptors to identify the sites of receptor phosphorylation and modification by DTSSP. Mutagenesis was then used to determine the effects of phosphorylation or modification by DTSSP on channel function. These studies identify the extent of P2X1 receptor phosphorylation and using lysine reactive compounds provide information on the accessibility of intracellular regions of the receptor as well as substantiate the importance of movement between adjacent subunits in determining agonist action.

## Materials and methods

### Protein purification and mass spectrometry protein analysis

Human P2X1 receptors with a C-terminal FLAGHis_6_ tag were isolated utilizing an Anti-FLAG® M2 Affinity agarose gel/column [method derived from (Roberts *et al*. 2012)]. Starting material of 5 × 175 cm^2^ flasks of human embryonic kidney 293 (HEK293) cells stably expressing human P2X1 FLAGHis_6_ tag and treated with phosphate-buffered saline alone (control), or with the addition of 100 μM DTSSP or 100 μM Dithio*bis*(sulfosuccinimidylpropionate) (DSP). The cells were lysed using buffer containing 150 mM NaCl, 40 mM Tris HCl (pH 7.5), 8 mM Tris base, 1% n-Octyl glucoside and protease inhibitor cocktail (Sigma, Poole, UK). Cell lysate was cleared by centrifugation (16 K for 15 min) and supernatant rolled overnight with 2 mL of Anti-FLAG® M2 Affinity agarose gel (Sigma). The agarose beads were placed in a column and flow through (FT) collected. Beads were washed four times with 25 mL of lysis buffer and a sample collected at each wash stage. Proteins were eluted with ten 1-mL aliquots of 0.1 mg/mL 3X FLAG peptide (E1 – E10) followed by three 1-mL aliquots of 0.1 M Glycine pH 3.5 (E11 – E13). Samples of fractions (10 μL) collected from each step were western blotted with anti-P2X1 antibody (Alomone, Jerusalem, Israel) and positive fractions (E2 – E8) pooled and concentrated using Amicon Ultra 430 000 MWCO centrifugal filters (Millipore, Billerica, MA, USA). Purified FLAGHis_6_ tagged human P2X1 receptor in some instances was treated with PNGase F to deglycosylate the receptor prior to being run on a 10% sodium dodecyl sulfate–polyacrylamide gel electrophoresis (SDS-PAGE) gel and stained using InstantBlue (Expedeon, Harston, UK). The stained band was excised, trypsin digested and analysed using mass spectrometry. Samples were analysed by liquid chromatography coupled with tandem mass spectrometry (LC-MS/MS) using either a 4000 Q-Trap (Applied Biosystems, Carlsbad, CA, USA) or an LTQ-Orbitrap-Velos (Thermo Scientific, Waltham, MA, USA) mass spectrometer. Database searched were performed using Mascot (version 2.2.04; Matrix Science Ltd., Boston, MA, USA) against a custom database containing the P2X1 sequence.

### Phosphopeptide enrichment

Purified human P2X1 receptors were excised from the polyacrylamide gel and washed three times for 15 min with 100 mM ammonium bicarbonate. Reduction and alkylation of cysteines were performed by addition of 10 mM dithiothreitol (DTT) in 50 mM ammonium bicarbonate at 60°C for 30 min followed by the addition of 100 mM iodoacetamide in 50 mM ammonium bicarbonate for 30 min in the dark. Gel slices were washed three times for 5 min with 50 mM ammonium bicarbonate containing 50% acetonitrile and incubated overnight at 37°C in 100 microlitres 50 mM ammonium bicarbonate containing 1 μg sequencing grade trypsin (Promega, Madison, WI, USA). After trypsin digestion the supernatant was removed and three gel slice washes combined with of 200 μL 50 mM ammonium bicarbonate containing 50% acetonitrile. The resulting peptide digest supernatant was concentrated using a Savant Speedvac (Thermo Scientific, Waltham, MA, USA) for approximately 1 h. The near dry pellet was resuspended in 40 μL 0.1% trifluoroacetic acid (TFA) and a small sample taken for MALDI mass spectrometry analysis.

The rest of the sample was mixed with 250 μL of immobilized metal ion affinity chromatography (IMAC) loading buffer (250 mM acetic acid, 30% acetonitrile) to which 60 μL of washed 50% beads (PHOS-Select™ Iron Affinity Gel; Sigma P9740) was added. The sample was rotated for 1 h at 22°C and washed twice with 200 μL IMAC load buffer followed by one 200 μL water wash. The sample was eluted from the beads with 100 μl of IMAC elution buffer (0.4 M Ammonium hydroxide, 30% acetonitrile). Following elution, the sample was reduced to near dryness using a speedvac and resuspended in 40 μL of 0.1% TFA. The phospho enriched peptides were eluted with 5 mg/mL 2,5-dihydroxybenzoic acid in 50% acetonitrile, 1% H_3_PO_4_ and a sample submitted for mass spectrometry.

### Western blotting

Western blotting was carried out for purified fractions to identify those that were enriched with P2X1 receptor protein. After addition of 2X SDS-PAGE sample buffer, the fraction samples were heated at 95°C for 5 min before loading on a 10% SDS-PAGE gel. Proteins were transferred to nitrocellulose, probed with P2X1 primary antibody (1 : 1000; Alomone Labs) followed by secondary goat anti-rabbit antibody (A6154, 1 : 3500 dilution; Sigma). Blots were developed using ECL Plus (GE Healthcare, Buckinghamshire, UK), and exposed to Hyperfilm film (GE Healthcare).

### P2X1 receptor site directed mutagenesis

Point mutations were made with the QuikChange™ mutagenesis kit (Stratagene, La Jolla, CA, USA) using a human P2X1 receptor plasmid as the template as described previously (Ennion *et al*. 2000). The S286C mutant had been made previously and was used as a template for some mutants, cysteine is not reported to be modified by DTSSP. Production of the correct mutations and absence of coding errors in the P2X1 mutant constructs was verified by DNA sequencing (Automated ABI Sequencing Service, University of Leicester).

### Two-electrode voltage clamp electrophysiology

Two-electrode voltage clamp recordings (at a holding potential of −60 mV) were carried out on cRNA injected oocytes using a GeneClamp 500B amplifier with a Digidata 1322 analog-to-digital converter and pClamp 8.2 acquisition software (Axon Instruments, Molecular Devices, Foster City, CA, USA) as previously described (Ennion *et al*. 2000). Native oocyte calcium activated chloride currents in response to P2X receptor stimulation were reduced by replacing 1.8 mM CaCl_2_ with 1.8 mM BaCl_2_ in the ND96 bath solution. ATP (Mg salt) was applied via a U-tube perfusion system. Oocytes expressing wildtype or mutant P2X1 receptors were treated for 30 min with or without 100 μM DTSSP and responses to 100 μM ATP tested.

### P2X1 receptor ATP-binding assay

^32^P 2-AzidoATP was used to label P2X1 receptors expressed in oocytes (Agboh *et al*. 2008). Oocytes were treated with or without 100 μM DTSSP for 30 min followed by washing with phosphate-buffered saline and then labelled using 1 μM ^32^P 2-AzidoATP and UV illumination. Densitometry analysis was performed on the resultant films with data corrected for background levels for each lane and expressed as a percentage of control untreated levels.

### Analysis

All data are shown as mean ± standard error of the mean. Significant differences were calculated by *t*-test or one-way analysis of variance followed by Dunnett's test for comparisons to control, a *p* value of < 0.05 considered significant. All statistics were carried out using Graphpad Prism 5 (GraphPad Software Inc., San Diego, CA, USA). *n* corresponds to the number of oocytes tested for electrophysiological data, and for biochemical studies experiments were repeated at least three times.

## Results

### Purification and mass spectrometry of human P2X1 receptors from HEK 293 cells

**Fig. 1:**
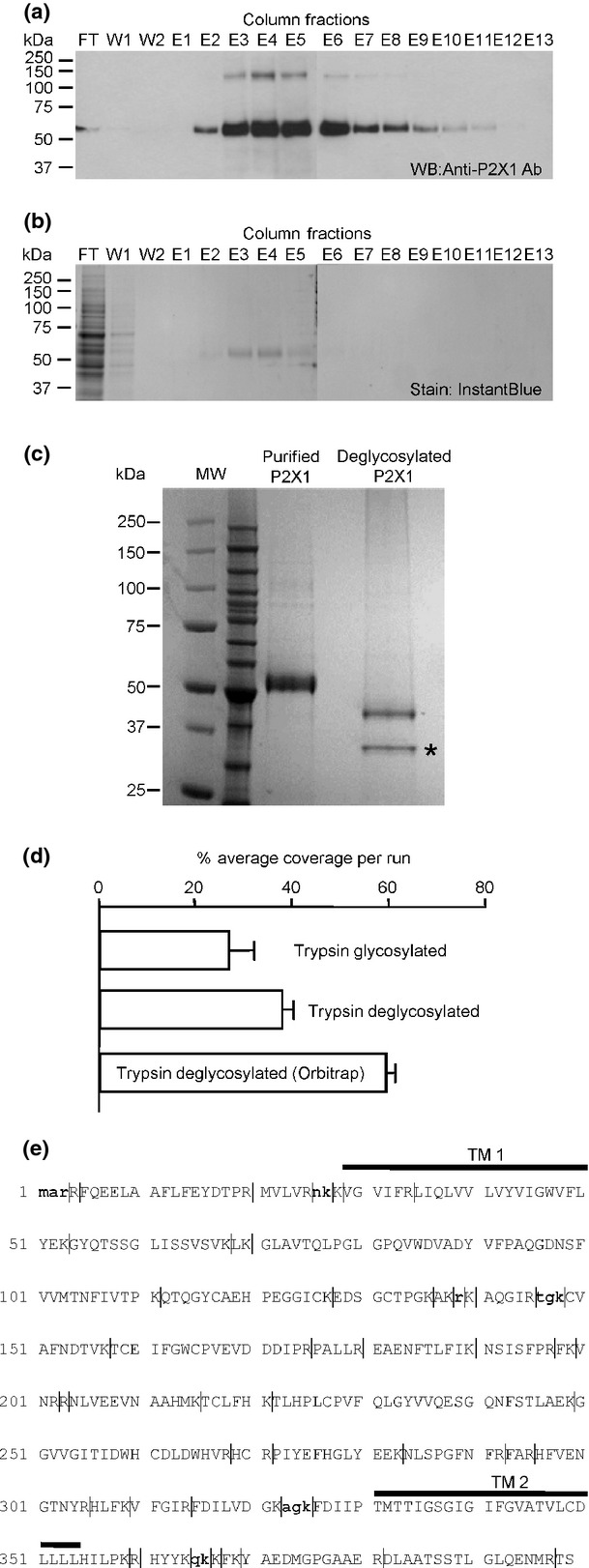
Purification and mass spectrometry analysis of the Human P2X1 receptor. (a) Anti-P2X1 receptor antibody western blot analysis of 3X FLAG peptide eluted fractions from anti-FLAG agarose beads. FT, flow through; W1–2, washes; E1–E10, FLAG peptide eluted fractions; E11–13, 0.1 M Glycine pH 3.5 eluted fractions. P2X1 protein of the correct mass is observed in fractions E2–E8. (b) InstantBlue (Expedeon) stained gel of eluted fractions from anti-FLAG agarose beads (lanes labelled as Fig. 1a). (c) Combined and concentrated fractions were run on a 10% sodium dodecyl sulfate–polyacrylamide gel electrophoresis (SDS-PAGE) and stained with InstantBlue (Expedeon). Clean purified FLAG tagged P2X1 protein can be observed along with PNGase F deglycosylated P2X1 receptor protein and the PNGase F protein (star indicates confirmed by mass spectrometry) (d) Identification of the P2X1 receptor with 26.8 ± 6% percentage coverage on single mass spectrometry runs was achieved with trypsin digest though other enzymes were tested (e.g. chymotrypsin, 11.2 ± 5% coverage, data not shown). Coverage of the P2X1 receptor protein was increased (26.8 ± 6% vs. 37.8 ± 3%) by deglycosylating the receptors with PNGase F. Use of Orbitrap versus Qtrap mass spectrometer increased mass accuracy and peptide identification increasing coverage even further on average to 59.2 ± 2%. (e) Human P2X1 receptor protein sequence showing the total coverage of all observed peptides (26 runs). Transmembrane regions 1 and 2 are highlighted with a bold line. Amino acid residues shown in bold lowercase were not observed on mass spectrometry of the P2X1 receptor protein most probably because their masses were below the limit of detection (∼500 Da) (Table S1). Other areas of predicted low mass were only observed as a result of partial digestion and therefore identified as part of a larger peptide mass. Vertical lines indicate sites for trypsin digestion at arginine and lysine residues.

Human P2X1 receptors were purified for mass spectrometry analysis using a C‐terminal FLAGHis_6_ tag. HEK293 cells stably expressing FLAGHis_6_ tagged human P2X1 receptors were lysed and anti‐FLAG agarose beads used to purify the receptor, positive fractions were identified by western blotting (Fig. 1a). The anti‐P2X1 receptor antibody labelled a predominant band of ∼ 55 kDa consistent with the P2X1 receptor (Ennion *et al*. 2000). Small amounts of P2X1 receptor were identified in the FT but were absent from the glycine washes. The P2X1 receptor was eluted from the column with FLAG peptide (fractions E2 – E8). To determine the level of purity of the sample a 10% SDS‐PAGE gel was also run and proteins stained with an InstantBlue Coomasie stain (Expedeon, Harston, UK) (Fig. 1b). As expected a wide range of proteins were detected in the FT and to a lesser extent in the first wash (W1). However, only a single band of ∼ 55 kDa was detected in elutions E3 – E6 consistent with purification of the P2X1 receptor. Positive fractions E2 – E8 were pooled and concentrated for mass spectrometry analysis. Concentrated pure human FLAGHis_6_ tagged P2X1 receptor was run on a 10% SDS‐PAGE gel and stained with InstantBlue coomasie stain (Fig. 1c). Following de‐glycosylation with PNGase F, the mass of the P2X1 receptor was reduced (Fig. 1c) consistent with a complete loss of glycosylation at all four known sites as observed previously (Roberts and Evans 2006).

In initial studies, purified FLAGHis_6_ tagged P2X1 receptor protein was excised from the 10% SDS-PAGE gel (stained with InstantBlue to indicate sufficient protein). The sample was treated with DTT and iodoacetamide to reduce disulphide bonds, digested with trypsin (cuts at Arg and Lys residues) and analysed by mass spectrometry. Coverage of the glycosylated human P2X1 receptor on single mass spectrometry runs averaged 26.8 ± 6% for trypsin digestion (Fig 1.d). Deglycosylation of the receptor protein with PNGase F increased coverage to 37.8 ± 3%. This improvement may be because of increasing the accessibility of the receptor to enzymatic digestion (Fig 1.d). Use of the more sensitive Thermo Orbitrap (compared with Qtrap 4000 used in initial studies) mass spectrometer further increased average coverage to 59.2 ± 2% average coverage per run. Some P2X1 receptor peptide fragments were never detected by the mass spectrometer most likely because of their small size or charge, and other peptides were observed infrequently (Table S1). Overall 96% of the receptor was observed over 26 runs of the mass spectrometer (Fig 1.e) with some peptides occurring more frequently than others (Table S1) and shows that mass spectrometry analysis may be a useful technique to investigate biochemical modification of the P2X1 receptor.

### Phosphorylation status of the human P2X1 receptor

**Fig. 2:**
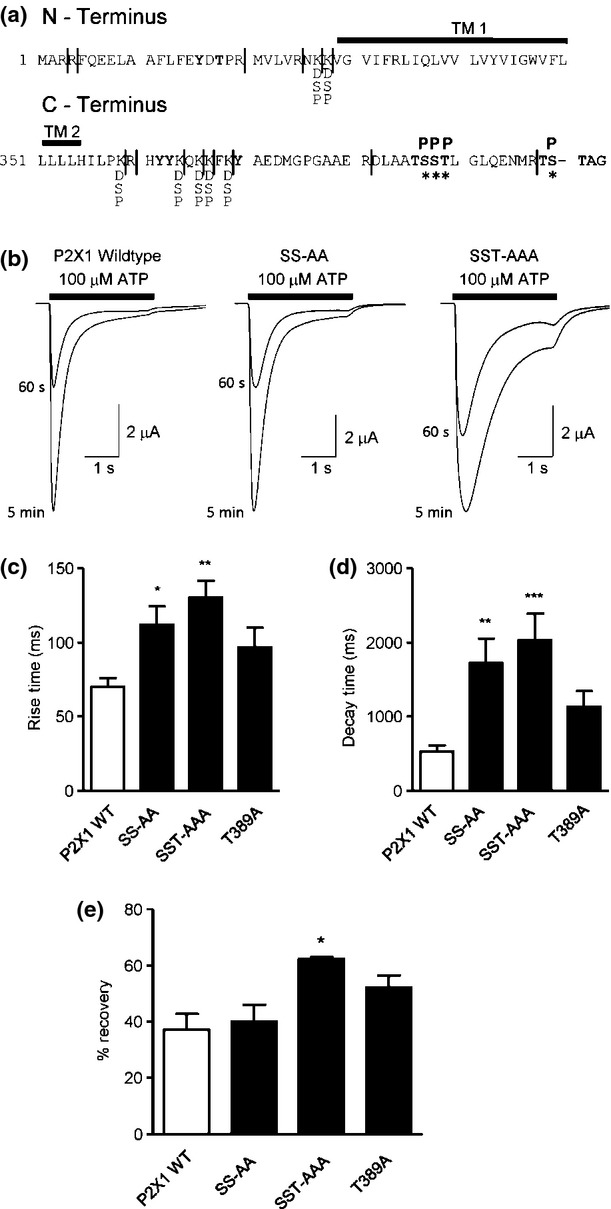
Phosphorylation status of the Human P2X1 receptor. (a) P2X1 receptor protein sequence of the N- and C-termini. Intracellular modifications made by membrane permeable Dithio*bis*(sulfosuccinimidylpropionate) (DSP) were identified by mass spectrometry and are indicated on the protein sequence. Membrane impermeable 3,3′-Dithio*bis* (sulfosuccinimidylpropionate) (DTSSP) modifications were only observed on extracellular P2X1 protein residues. DSP modification highlights the lipid/transmembrane boundaries with K27 and K28 modified showing accessibility. *In silico* analysis of the N-terminal protein sequence reveals residues Y16 and T18 as potential phosphorylation sites (bold type). Mass spectrometry analysis did not show modification at these residues even after enriching for phosphorylated peptides. *In silico* analysis of the C-terminal protein sequence reveals tyrosine residues Y362, 363 and 370 and serine and threonine residues 386–389 and 398–399 as potential phosphorylation sites (bold type). Phosphorylation was initially observed at residues S387 and S388 and residues S388, T389 and S399 were identified on mass spectrometry runs enriched for phosphorylated peptides (P *). These residues were the only phosphorylated residues observed on mass spectrometry of the P2X1 receptor protein. (b) ATP evoked currents (period indicated by bar) from P2X1 wildtype and mutant receptors (S387A and S388A – SS-AA, S387A, S388A and T389A – SST-AAA). Traces show reproducible response evoked at a 5-minute interval and level of recovery from desensitization at 60 s between ATP applications. (c, d) Time to peak (10–90%) and decay time (100–50%) of currents evoked by 100 μM ATP for wildtype and mutant P2X1 receptors. (e) Recovery from desensitization at 60 s for wildtype and mutant P2X1 receptors. *p < 0.05, **p < 0.01, ***p < 0.001, (*n* = 4–6).

We have shown previously using radiolabelled ^32^P incorporation that the human P2X1 receptor is phosphorylated (Vial *et al*. 2004); however, which of the intracellular threonine (3), serine (4) and tyrosine (4) residues that could potentially be modified was not determined. We have now used LC-MS/MS to determine which residues can be phosphorylated. Amino acid modifications corresponding to phosphorylation were only detected twice corresponding to residues S387 and S388 (1 phosphorylated fragment for each out of identified fragments from 18 positive runs). Fragments corresponding to non-phosphorylated residues were detected for Y16 (15 runs), T18 (15 runs), Y362/Y363 (5 runs), Y370 (19 runs), T386 (18 runs), T389(18 runs), T398 (1 run) and S399 (1 run). Phospho-protein fragments are often difficult to detect and so we used a protocol utilizing an iron bead complex to enrich negatively charged peptides and hence phosphopeptides. Using this technique, phosphoprotein fragments were detected in three of three isolations. Analysis of these fragments showed phosphorylation of residues S388 (2 of 3 runs), T389 (2 of 3 runs) and S399 (1 of 3 runs) (Fig 2.a). S399 would normally be the terminating amino acid of the P2X1 receptor, but a C-terminal FLAGHis_6_ tag was added after this to aid purification. We are not aware of any examples of where a final C-terminal amino acid is phosphorylated in other proteins, and we suggest that the phosphorylation that was detected at S399 results from the addition of the FlagHis_6_ tag and that S399 is not phosphorylated in the native P2X1 receptor. Peptide fragments containing the potential phosphorylation sites Y16, T18, Y370, T386 and T398 were identified in 3/3 enrichment runs but no phosphorylation was detected. Residues Y362/363 were not detected in the enriched runs, this may result from incomplete digestion of the samples and therefore inclusion of additional positively charged adjacent arginine or lysine residues that would not bind to the iron bead complex.

To investigate the contribution of phosphorylation to P2X1 receptor properties the alanine mutants T389A, double mutant S387A-S388A (SS-AA) and triple mutant S387A, S388A and T389A (SST-AAA) were generated and characterized (Fig 2.b–e). At wild type human P2X1 receptors ATP (100 μM) evoked inward currents that rapidly declined during continued agonist application (referred to as desensitization). The T389A mutation had no effect on peak current amplitude, the time course of the response (10–90% rise time or time to 50% decay), or the recovery from desensitization (measured as% recovery of peak current with 1 minute between agonist applications) indicating that phosphorylation at this position is not essential for normal channel function. Removal of sites of serine phosphorylation with the double mutants S387A-S388A gave a modest ∼25% increase in peak current amplitude and a 3.3-fold slowing in desensitization of the ATP evoked current with no effect on recovery from desensitization. The triple mutant S387A-S388A-T389A also had increased peak current amplitudes (∼30%) a slowing in current time course and a 20% increase in recovery from desensitization. These results indicate that phosphorylation of the P2X1 receptor is not essential for normal channel function, and that alanine mutation to remove phosphorylated serine and threonine residues modified channel properties.

### Accessibility of intracellular lysine residues revealed by DSP modification

The intracellular carboxyl terminus of P2X receptors contains a conserved trafficking motif that contains lysine residues (Chaumont *et al*. 2004), some of which have been suggested to be involved in phospholipid binding (Fujiwara and Kubo 2006; Bernier *et al*. 2008a). Residues in the intracellular amino terminus have also been suggested to be involved in regulation of channel gating through interactions with the transmembrane segments and the carboxy terminus (Allsopp and Evans 2011). However, at present there is little information about the structure of these regions as the intracellular amino and carboxyl termini of the zebrafish P2X4 receptor were removed to aid crystallization (Kawate *et al*. 2009; Hattori and Gouaux 2012). To gain insight into the accessibility of the intracellular domains, we used the membrane permeant amine reactive compound DSP to determine whether the intracellular lysine residues were accessible. Following DSP treatment (100 μM for 30 min), the cells were lysed in a TRIS buffer that quenches the activity of the DSP, before purification of the P2X1 receptor. This protocol therefore can be used to demonstrate the accessibility of intracellular lysine residues in their native state in the HEK293 cell membrane. Mass spectrometry of purified P2X1 receptors showed that all the intracellular lysine residues were accessible and modified by DSP (Fig 2.a). The accessibility of these lysine residues adjacent to the first transmembrane region (residues 27, 28) and five amino acids from the second transmembrane region (residue 359), and around the conserved trafficking motif (residues 364, 366, 367, 369) to modification by DSP demonstrates that in the native cellular environment, the residues are accessible in at least one subunit of the receptor at some point.

### Mass spectrometry identification of site of DTSSP modification of the Human P2X1 receptor

**Fig. 3:**
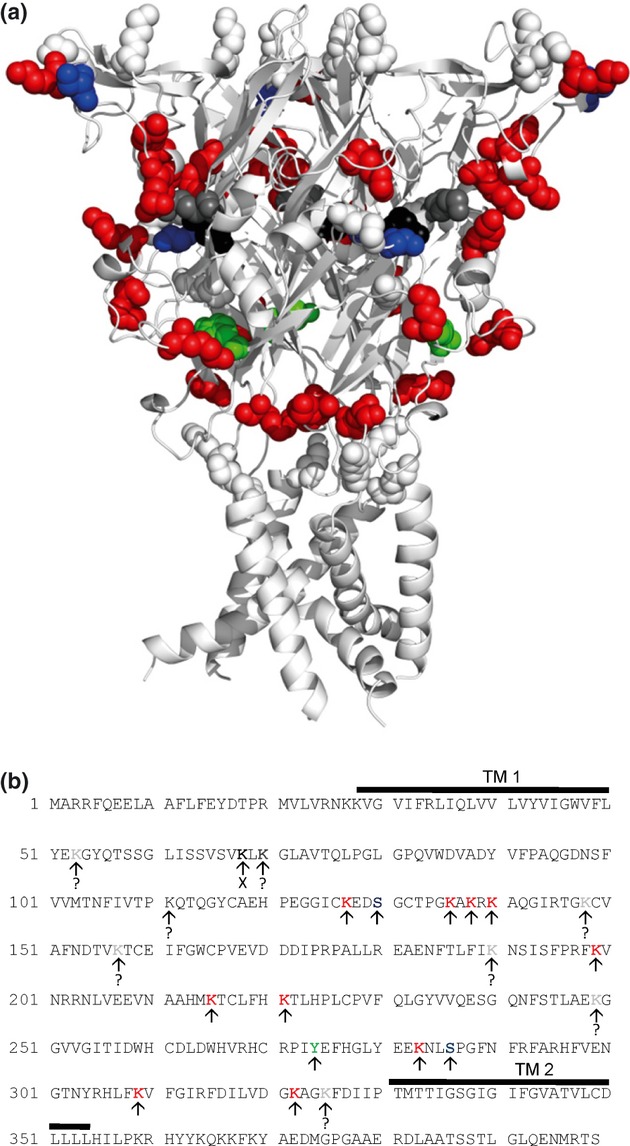
Localization of 3,3′-Dithio*bis* (sulfosuccinimidylpropionate) (DTSSP) modification of the human P2X1 receptor. (a) P2X1 homology model (based on zP2X4 crystal structure model – Kawate *et al*. 2009) depicted as a trimeric cartoon. Lysine residues modified DTSSP and identified by mass spectrometry are indicated in red. Lysine residue K70 that was not observed in any mass spectrometry runs is shown in dark grey. Residues observed and not modified by DTSSP in some runs are shown in grey. Residue K68 that was not observed to be modified by DTSSP (11/11) is shown in black. The serine residues 130, 286 (blue spheres) and tyrosine 274 (green spheres) were also modified by DTSSP. (b) Human P2X1 protein sequence with DTSSP modification of the P2X1 receptor marked as observed from mass spectrometry data. Transmembrane regions 1 and 2 are highlighted with a bold line. Residues are coloured as indicated in panel (a). Arrows indicate residues modified by DTSSP, an arrow with a cross show a residue that was not modified by DTSSP, and arrows with a question mark where it remains unknown either because of non-coverage of the protein sequence or modification not detected. Note that all the DTSSP modifications occur in the extracellular region.

The homo-bifunctional membrane-impermeant cross-linker DTSSP has previously been used to show the trimeric assembly of P2X receptors; however, its site(s) of action were not determined. DTSSP reaction with the P2X1 receptor results in a thioacyl or thioacyl iodo modification of the receptor that can be detected by mass spectrometry. Of the 19 extracellular lysine residues, 10 were observed to be modified by DTSSP (K127, K136, K138, K140, K199, K215, K221, K283, K309 and K322) and residue K68 was only detected in the native state in 11/11 runs indicating that this residue does not bind DTSSP. Peptides corresponding to the remaining eight lysine residues were only observed in one to four of the 11 runs and only the native un-modified lysine was detected; thus, for these eight residues, it remains unknown whether they are modified (Fig. 3; Table). Surprisingly DTSSP modification was also observed at residues S130, S286 and Y274, similar labelling of serine and tyrosine residues by DTSSP has been reported previously (Swaim *et al*. 2004). Mapping the sites of DTSSP modification onto a human P2X1 receptor homology model (Allsopp *et al*. 2011) shows that these residues are all in accessible positions on the surface of the receptor (Fig 3.) and provides independent support for the validity of the P2X1 receptor homology model.

**Table 1:** Molecular modelling of DTSSP modification of the human P2X1 receptor

	Residues within 12 angstroms (based on P2X model)	DTSSP modification (11 runs)
K53	322 (12.8), 325 (12.9)	NO (3)/Unknown (8)
K68	-	NO (11)
K70	**140 (8.2)**, 215 (10.4), **286 (10.2)**, **309 (11.1)**	Unknown (11)
K111	None	NO (3)/Unknown (8)
K127	130 (7.9), 157 (8.4)	YES (5)/NO (6)
K136	138 (11.2), 140 (7.7)	YES (4)/NO (4)
K138	136 (11.2), 140 (12.4)	YES (4)/Unknown (7)
K140	**70 (8.2)**, 136 (7.7), 138 (12.4), **215 (12.3)**	YES (6)/Unknown (5)
K148	None	NO (1)/Unknown (10)
K157	127 (8.4), 130 (12)	NO (3)/Unknown (8)
K190	274 (12), **283 (8.7)**, **286 (6.7)**	NO (1)/Unknown (10)
K199	325 (10.6), **221 (12.5)**	YES (1)/NO (2)
K215	70 (10.4), **140 (12.3)**	YES (7)/NO (3)
K221	**199 (12.5)**, 274 (11.1)	YES (4)/NO (4)
K249	283 (12.8), 286 (8.6), 309 (11)	NO (2)/Unknown (9)
K283	**190 (8.7)**, 249 (12.8), 286 (12.3)	YES (2)/NO (6)
K309	**70 (11.1)**, 249 (11), 286 (6.7)	YES (2)/NO (2)
K322	53 (12.8), **322 (8.1)**	YES (4)/NO (6)
K325	53 (12.9), 199 (10.6)	NO (4)/Unknown (7)
S130	127 (7.9), 157 (12)	YES (1)/NO (10)
S286	**70 (10.2)**, **190 (6.7)**, 249 (8.6), 283 (12.3), 309 (6.7)	YES (5)/NO (6)
Y274	190 (12), 221 (11.1)	YES (2)/NO (9)

Utilizing the zP2X4 closed state receptor model (Kawate *et al*. 2009), 19 extracellular lysine residues and also three non-lysine residues shown to be modified by 3,3′-Dithio*bis* (sulfosuccinimidylpropionate) (DTSSP) on mass spectrometry analysis were mapped. Potential binding partners were identified that may be responsible for DTSSP inhibition and/or formation of dimers/trimers (dimensions were calculated in angstroms as a measurement made of the P2X1 homology model of the zP2X4 crystal structure in PyMol). Under non-reducing conditions, no peptides were observed on ms coupled together by non-reduced DTSSP. Residue K68 was not observed to bind DTSSP on mass spectrometry analysis and was therefore not analysed. Several residues were noted to have possible binding partners and were the subject of further investigations using site directed mutagenesis. Residues marked in bold print are from separate receptor subunits (formation of dimer/trimers or possible inhibition of intersubunit movement), whereas those marked in normal print may possibly exhibit allosteric or binding site inhibition of receptor function by intrasubunit modifications. Analysis of mass spectrometry data of 11 runs identified DTSSP modified residues on observed peptides (YES) or no modification on an observed peptide (NO). Where the peptide was not observed for a particular residue in a run the result was declared as unknown. Where there was a mixed yes/no result the remainder were unknown (11 runs total).

As DTSSP is a bifunctional compound it would be theoretically possible to detect coupled peptides that could account for the cross-linking of the P2X1 receptor to form a trimer. DTSSP can be cleaved in two by reducing agents, and therefore, initially we purified the P2X1 receptor and prepared samples for mass spectrometry under non-reducing conditions. However, under these conditions there was a drastic reduction in coverage of the receptor (12.3 ± 3.9%) on mass spectrometry analysis, and no coupled peptides were observed with DTSSP modification for non-reduced receptor protein. We were therefore not able to determine directly any DTSSP cross-links between subunits.

### DTSSP inhibits P2X1 receptor responses

**Fig. 4:**
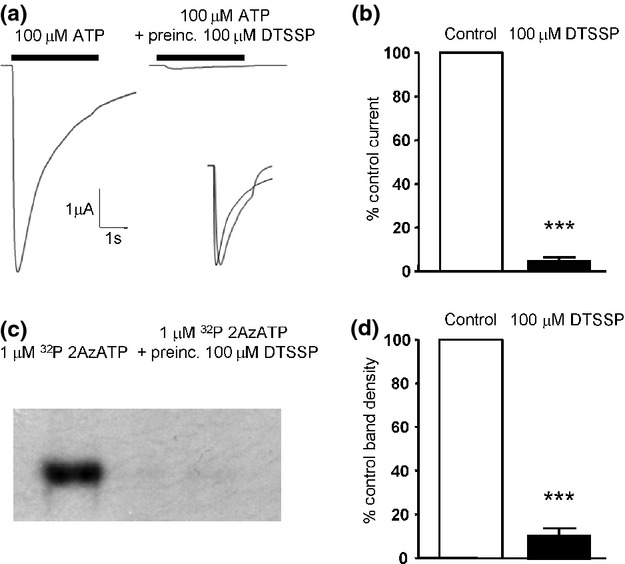
Effect of 3,3′-Dithio*bis* (sulfosuccinimidylpropionate) (DTSSP) modification on Human P2X1 receptor function. (a) Application of 100 μM ATP to *Xenopus* oocytes expressing P2X1 wildtype receptors evoked a large inward current recorded by two electrode voltage clamp. Pre-incubation with 100 μM DTSSP for 30 min almost abolished responses. Inset traces are representative of normalized responses to 100 μM ATP in the presence or absence of 100 μM DTSSP indicating no significant change in the current time course. (b) Pooled electrophysiology data depicting the decrease in channel function post-DTSSP treatment. (c) P2X1 receptor ATP-binding site analysis utilizing *uv* cross-linked ^32^P 2Azido ATP (2AzATP) shows a marked reduction in radioactivity of the P2X1 receptor protein band following pre-treatment with 100 μM DTSSP. (d) Pooled densitometry data collected from autoradiography of the DTSSP treated and non-treated 2AzATP radioactive P2X1 receptor bands (*n* = 4) ***p < 0.001.

The mass spectrometry studies demonstrated that DTSSP can bind to several sites in the extracellular loop of the receptor, and we have now determined whether this affects receptor function. In this study, we tested the effects of DTSSP treatment on human P2X1 receptors. ATP (100 μM) evoked transient P2X receptor inward currents (6767 ± 1057 nA) that decayed during the continued presence of agonist as reported previously (Ennion *et al*. 2000). Following pre-incubation with DTSSP (100 μM 30 min), the amplitude of ATP evoked currents was reduced by > 95% (Fig 4.a and b). However, there was no effect on the time course of the P2X1 receptor current (Time from peak current to 50% decay for control 526 ± 85 ms and following DTSSP 383 ± 66 ms *n* = 6.9).

To determine whether this reduction resulted from an effect on agonist binding to the P2X1 receptor, we used a radioactive 2-Azido ATP-binding assay (Roberts and Evans 2007). In control oocytes expressing P2X1 receptors ^32^P 2-Azido ATP (1 μM) binding was detected by autoradiography (Fig 4.c and d) as reported previously (Roberts and Evans 2007). The P2X1 protein band radioactivity was reduced to 10.7 ± 3% of control (*n* = 5) following pre-treatment with 100 μM DTSSP (Fig 4.c and d). These results show that DTSSP inhibits agonist binding to the P2X1 receptor.

### Effects of DTSSP at P2X1 receptor mutants

**Fig. 5:**
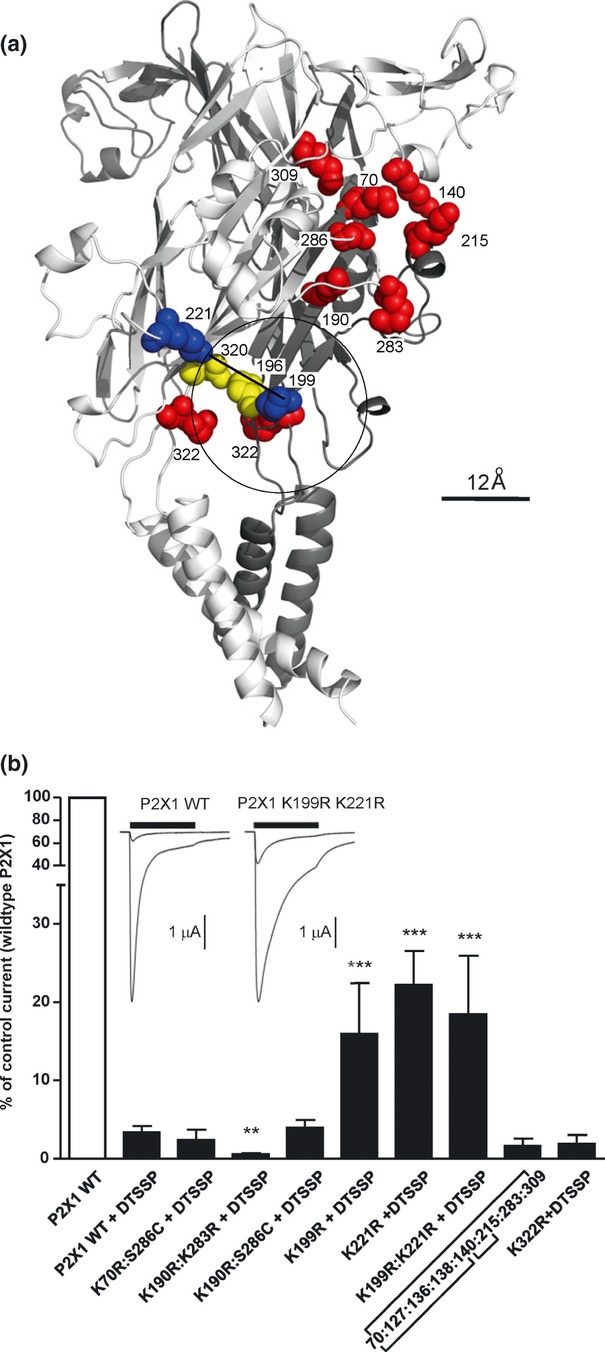
Site directed mutagenesis of human P2X1 receptor to discover the molecular basis for 3,3′-Dithio*bis* (sulfosuccinimidylpropionate) (DTSSP) inhibition. (a) Cartoon representation of P2X1 receptor structure highlighting the residues K199 and K221 (blue spheres) positioned on different subunits approximately 12 angstroms apart. The diameter of the circle shown is approximately 24 angstroms. Residues in yellow correspond to positions 196 and 320 in adjacent subunits where introduced cysteine residues form a disulphide bond and inhibit channel activation. Red spheres correspond to lysine residues that when mutated had no effect on DTSSP inhibition of ATP evoked responses. (b) Combining mass spectrometry and crystal structure to map possible cross-linked pairs, multiple mutations were designed to discover the residues responsible for DTSSP inhibition and cross-linking. Residues 70 : 140; 70 : 309; 70 : 286; 140 : 215; 190 : 283; 190 : 286; 199 : 221 and 322 : 322 are all within 12 angstroms and on separate P2X1 subunits and therefore possible candidates for causing functional inhibition and dimer/trimer formation with DTSSP. Only double mutant K199R:K221R showed a significant reduction of DTSSP inhibition also reflected in the single mutants K199R and K221R (*n* = 3–25). No mutations were observed to disrupt the DTSSP formation of dimers/trimers on western blot (data not shown). Inset example trace data for human P2X1 wildtype and P2X1 double mutant K199R: K221R in the presence and absence of 100 μM DTSSP **p < 0.01, ***p < 0.001.

The reduction of the amplitude of ATP evoked currents, with no effect on the time course of the response by DTSSP was similar to that we have recently reported for double cysteine mutants between subunits that restricted conformational change (Roberts *et al*. 2012). Mapping the DTSSP modification data to the P2X1 receptor homology model indicates several lysine residues in adjacent subunits that are within 12 angstrom of each other (distance between the reactive groups of DTSSP), that is, 70 : 140; 70 : 309; 70 : 286; 140 : 215; 190 : 283; 190 : 286; 199 : 221 and 322 : 322. To test whether DTSSP cross-linking between adjacent subunits contributed to the reduction in ATP evoked currents, we generated a series of double lysine mutants as well as a mutant removing several lysine residues around the ATP-binding pocket (mutants tested K70R:S286C; K190R:K283R; K190R:S286C; K199R:K221R; K70R:K127R:K136R:K138R:K140R:K215R:K283R:K309R and K322R). Mutating the lysine residues around the ATP-binding pocket so that DTSSP could no longer bind had no effect on the inhibition by DTSSP (Fig 5.). Of the mutants tested, only the K199RK221R mutant showed a reduced level of inhibition by DTSSP compared with the WT P2X1 receptor (*p* = 0.001). To further address the change in inhibition, we tested the effects of DTSSP on the single mutants K199R and K221R and these showed the same effect as the double mutant (Fig 5.). The fact that there is no additive effect of combining the single mutants suggests that it is the DTSSP cross-linking between the subunits at these residues that restrains channel conformational change and inhibits ATP evoked responses. This raises the possibility that movement between subunits is essential for high affinity binding to the receptor.

## Discussion

The isolation of tagged recombinant P2X receptors has been used previously to identify interacting regulatory proteins (Kim *et al*. 2001; Lalo *et al*. 2011), look at agonist binding (Chen *et al*. 2000), and in single particle transmission electron microscopy studies shown marked conformational changes in the receptor on agonist binding (Roberts *et al*. 2012). In this study, we used a combination of protein purification, amine reactive reagents, mass spectrometry and mutagenesis to determine the phosphorylation status of the human P2X1 receptor, accessibility of intracellular lysine residues, provided direct support for the receptor homology model and conformational movements that are important for receptor function.

The detection of multiple sites of P2X1 receptor phosphorylation by mass spectrometry is consistent with our previous work showing ^32^P incorporation into the receptor (Vial *et al*. 2004). This study has now identified residues that are basally phosphorylated and could potentially contribute to channel function as well as residues where any phosphorylation was below the limit of detection. Analysis of the P2X1 receptor with bioinformatic programs indicates Y16, Y363 and Y370 as potential sites for tyrosine phosphorylation. In this study intracellular tyrosine residues 16 and 370 were detected by mass spectrometry but they were not phosphorylated. This is consistent with a study using anti-phosphotyrosine antibodies (Toth-Zsamboki *et al*. 2002). In addition previous mutant studies demonstrated that individual tyrosines, and hence potential phosphotyrosines, are not essential for channel function. However, the properties of the mutant were dependent on the amino acid substitution. At Y363 the aromatic group is important, whilst the polar group is more important for activity at Y16, Y362 and Y370 as responses could be evoked at cysteine point mutants but not phenylalanine (Toth-Zsamboki *et al*. 2002; Wen and Evans 2009, 2011). However, in all cases with the exception of Y370C, there was a reduction in peak current amplitude that did not result from an effect on surface expression of the receptor. The current results support and extend previous work and strongly suggest that the P2X1 receptor is most likely not constitutively regulated by tyrosine phosphorylation.

The role of the conserved consensus protein kinase C motif TXK/R in the amino terminus has been the subject of numerous papers with some providing evidence of phosphorylation in P2X1 and P2X2 receptors (Liu *et al*. 2003) (Boue-Grabot *et al*. 2000). The site is of particular functional importance as mutation of the threonine leads to an increased rate of desensitization and reduction in peak current amplitude (Boue-Grabot *et al*. 2000; Ennion and Evans, 2002). Similarly, the lack of detection of phosphorylation of the conserved Thr18 residues in this study is consistent with previous work that the conserved consensus protein kinase C can be disrupted with no effect on time course (Jiang *et al*. 2001; Wen and Evans 2009). However, it should be noted that the phenotype of mutants in this region before the first transmembrane segment is highly variable depending on the substitution (Jiang *et al*. 2001; Liu *et al*. 2003; Wen and Evans 2009)(Boue-Grabot *et al*. 2000; Ennion and Evans, 2002). We have recently shown that residues around the consensus protein kinase C site can exert a dominant role over the time course of P2X1 and P2X2 receptor currents (Allsopp and Evans 2011). Taken together, this suggests that the amino acid composition of this region of the receptor, and not phosphorylation, that is important for channel function.

In this study, we show that P2X1 receptor is basally phosphorylated at residues S387, S388 and T389. These residues do not correspond to any consensus sequences for phosphorylation in current databases so the identification of the kinase(s) responsible remain to be determined. Alanine mutants to remove phosphorylation from the P2X1 receptor resulted in a small/modest increase in current amplitude, slowing the in the time course of the response and an increase in recovery from desensitization. We have shown previously that recovery from desensitization is dependent on the integrity of the intracellular environment (Lewis and Evans 2000). The increased speed in recovery from desensitization for the phosphorylation deficient triple mutant raises the possibility that de-phosphorylation of the receptor may contribute to recovery from desensitization (and dialysis of phosphatases in the whole cell patch clamp recording configuration contributes to receptor run down).

The intracellular regions of the P2X receptor have important regulatory roles; however, at present there is only structural information for a fragment of the C-terminus of the P2X4 receptor (Royle *et al*. 2005). In this study, we show that lysine residues 27 and 28 can be modified by membrane permeant DSP. These residues are just before the first transmembrane segment. As the first transmembrane segment does not line the P2X receptor channel this suggests that Lys^27,28^ are exposed to the intracellular aqueous environment and are at the interface with the lipid bilayer. DSP modification also shows that all the lysine residues in the carboxyl terminal (within ∼15 amino acids of the second transmembrane segment) are accessible. 4/5 of these lysine residues are associated with a conserved YXXXK trafficking motif that stabilizes P2X receptors at the cell surface (Chaumont *et al*. 2004). The lysine residues in this region have been shown to be involved in phospholipid binding to the carboxyl terminal P2X1 and P2X2 receptors (Fujiwara and Kubo 2006; Bernier *et al*. 2008b). The mass spectrometry analysis does not give an indication of stoichiometry of DSP binding. However, it does show that at least one of these carboxyl terminal regions is accessible at some point and indicates that either phospholipid binding is a dynamic process or not all subunits have phospholipid bound at once. These results also support our recent mutagenesis studies that suggested that this region is important for the binding of a regulatory factor associated with potentiation of P2X1 receptors by phorbol esters and phospholipase C G-protein coupled receptor signalling (Wen and Evans 2011).

DTSSP was initially used in the study of P2X receptors to demonstrate that P2X1 receptors form as trimers (Nicke *et al*. 1998). The identification of the sites of DTSSP binding by mass spectrometry in this study has identified the accessibility of a range of residues in the extracellular region of the receptor. Mapping of these residues onto the human P2X1 receptor homology model built using the zebrafish P2X4 receptor structure shows that they are all accessible on the receptor surface and provides direct support for the model. ATP evoked P2X1 receptor currents result from agonist binding and the subsequent gating of the receptor channel. The equivalent ∼ 90% reduction by DTSSP of agonist binding and evoked currents suggests that DTSSP acts primarily to block the initial binding step. It seems likely that DTSSP binding to residues within/close to the ATP-binding exerts steric effects to result in reduced ATP binding. However, the mutants K199R and K221R and the double mutant K199RK221R that reduced the extent of DTSSP inhibition are too far away from the agonist binding site for DTSSP to have a direct effect. The equivalent effects of the single and double mutation suggest that DTSSP acts to cross-link the residues K199 and K221 from adjacent subunits (either single mutant would block this cross-linking) and constrain conformational movements in the receptor. This would be consistent with the inhibitory effects of introduced disulphide bonds (196C and 320C) at the interface between P2X1 receptor subunits (Roberts *et al*. 2012). The recent crystal structures of the P2X4 receptor suggest that agonist induced conformational changes are greatest in this part of the receptor following agonist binding (Hattori and Gouaux 2012). This suggests that extensive conformational changes in the receptor are associated with the agonist binding step.

In summary, in this study, we have provided new information regarding the intracellular regions of the P2X1 receptor showing the phosphorylation status of the human P2X1 receptor and demonstrated the accessibility of regions close to the transmembrane segments. In addition, analysis of the actions of DTSSP has provided further functional validation of our P2X1 receptor homology model and the importance of conformational change for channel activation.

## Abbreviations

DSP: Dithio*bis*(sulfosuccinimidylpropionate)
DTSSP: 3,3′-Dithio*bis* (sulfosuccinimidylpropionate)
DTT: dithiothreitol
FT: flow through
HEK293: human embryonic kidney 293
IMAC: immobilized metal ion affinity chromatography
LC-MS/MS: liquid chromatography coupled with tandem mass spectrometry
SDS-PAGE: sodium dodecyl sulfate–polyacrylamide gel electrophoresis
TFA: trifluoroacetic acid

## Supporting Information

Table S1 Predicted trypsin digest of the P2X1 receptor protein and mass spectrometry observed peptides.

As a service to our authors and readers, this journal provides supporting information supplied by the authors. Such materials are peer-reviewed and may be re-organized for online delivery, but are not copy-edited or typeset. Technical support issues arising from supporting information (other than missing files) should be addressed to the authors.
