# Supplementary material for: Mass spectrometry analysis of human P2X1 receptors; insight into phosphorylation, modelling and conformational changes
Source: J Neurochem. 2012 Oct 11;123(5):725–35. doi: 10.1111/jnc.12012 (PMC3532615; doi:10.1111/jnc.12012)
Supplement: Table S1 — Predicted trypsin digest of the P2X1 receptor protein and mass spectrometry observed peptides. [file jnc0123-0725-SD1.pdf]

**Position of cleavage**	**Peptide sequence**	**Peptide mass [Da]**	**MS observed**	**of 22 runs**	**trypsin glycosyl**	**trypsin deglycosyl**	**Trypsin deglycosyl Orbitrap**
3	MAR	376.474	**x**				
4	R	174.203	yes	15	2	3	10
20	FQEELAAFLFEYDTPR	1976.173	yes	17	3	4	10
25	MVLVR	616.82	yes	1			1
27	NK	260.293	**x**				
28	K	146.189	yes	6			6
34	VGVIFR	689.856	yes	10			10
53	LIQLVVLVYVIGWVFLYEK	2294.849	yes	2			2
68	GYQTSSGLISSVSVK	1512.68	yes	18	2	7	9
70	LK	259.349	yes	1			1
111	GLAVTQLPGLGPQVWDVADYVFPAQGDNSFVVMTNFIVTPK	4393.037	yes	3			3
127	QTQGYCAEHPEGGICK	1720.892	yes	17	2	6	9
136	EDSGCTPGK	892.936	yes	7			7
138	AK	217.268	yes	1			1
139	R	174.203	**x**				
140	K	146.189	yes	3			3
145	AQGIR	543.624	yes	3			3
148	TGK	304.346	**x**				
157	CVAFNDTVK	996.146	yes	7		5	2
175	TCEIFGWCPVEVDDDIPR	2094.341	yes	17	2	7	8
180	PALLR	568.717	yes	17	2	7	8
190	EAENFTLFIK	1211.38	yes	9		5	4
197	NSISFPR	819.916	yes	14	2	2	10
199	FK	293.366	yes	1			1
202	VNR	387.439	yes	1			1
203	R	174.203	yes	14	1	4	9
215	NLVEEVNAAHMK	1354.544	yes	20	2	8	10
221	TCLFHK	747.91	yes	11	1	1	9
249	TLHPLCPVFQLGYVVQESGQNFSTLAEK	3106.543	yes	9		3	6
268	GGVVGITIDWHCDLDWHVR	2178.453	yes	7	1	1	5
271	HCR	414.483	yes	14	3	3	8
283	PIYEFHGLYEEK	1524.693	yes	14	3	3	8
292	NLSPGFNFR	1051.17	yes	22	4	8	10
295	FAR	392.458	yes	1			1
305	HFVENGTNYR	1236.309	yes	11		7	4
309	HLFK	543.666	yes	6			6
314	VFGIR	590.723	yes	6			6
322	FDILVDGK	906.046	yes	18	1	7	10
325	AGK	274.32	**x**				
359	FDIIPTMTTIGSGIGIFGVATVLCDLLLLHILPK	3583.389	yes	4			4
360	R	174.203	yes	7	2	1	4
364	HYYK	609.682	yes	7	2	1	4
366	QK	274.32	**x**				
367	K	146.189	yes	2			2
369	FK	293.366	yes	6			6
381	YAEDMGPGAAER	1266.348	yes	22	4	8	10
397	DLAATSSTLGLQENMR	1706.888	yes	22	4	8	10
399	TS	206.199	**x**				
